# The Influence of Outer Membrane Protein on Ampicillin Resistance of *Vibrio parahaemolyticus*

**DOI:** 10.1155/2023/8079091

**Published:** 2023-01-13

**Authors:** Xiangyu Meng, Danyang Huang, Qing Zhou, Fan Ji, Xin Tan, Jianli Wang, Xiaoyuan Wang

**Affiliations:** ^1^School of Biotechnology, Jiangnan University, Wuxi 214122, Jiangsu, China; ^2^School of Food Science and Technology, Jiangnan University, Wuxi 214122, China; ^3^State Key Laboratory of Food Science and Technology, Jiangnan University, Wuxi 214122, China

## Abstract

The antibiotic resistance of the food-borne pathogen *Vibrio parahaemolyticus* has attracted researchers' attention in recent years, but its molecular mechanism remains poorly understood. In this study, 7 genes encoding outer membrane proteins (OMPs) were individually deleted in *V. parahaemolyticus* ATCC33846, and the resistance of these 7 mutants to 14 antibiotics was investigated. The results revealed that the resistance of the 7 mutants to ampicillin was significantly increased. Further exploration of 20-gene transcription changes by real time-qPCR (RT-qPCR) demonstrated that the higher ampicillin resistance might be attributed to the expression of *β*-lactamase and reduced peptidoglycan (PG) synthesis activity through reduced transcription of penicillin-binding proteins (PBPs), increased transcription of l,d-transpeptidases, downregulated d,d-carboxypeptidase, and alanine deficiency. This study provides a new perspective on ampicillin resistance in OMP mutants with respect to PG synthesis.

## 1. Introduction

*Vibrio parahaemolyticus* is a Gram-negative bacterium causing intestinal infections. It is commonly found in seafood, such as blood clams and shrimp [1], causing significant economic losses as an aquatic pathogen [2]. Due to the overuse of antibiotics, multidrug-resistant *V. parahaemolyticus* has been isolated in recent years. The strains have demonstrated resistance to ampicillin, cefazolin, penicillin, and so on [3].

Studies investigating the mechanism of ampicillin resistance mainly focus on three points: drug permeation, peptidoglycan (PG) synthesis, and *β*-lactamase. In *Neisseria meningitidis*, a single point mutation in the porin PorB can strongly affect the binding and permeation of *β*-lactam antibiotics [4]. Affinity binding studies of four transformants revealed decreased affinity of PBP4 for ampicillin [5]. In *Enterococcus faecium*, l,d-transpeptidase-mediated resistance may emerge in various pathogens [6]. In *V. parahaemolyticus*, a novel class A carbenicillin-hydrolyzing *β*-lactamase, *bla* (CARB-17), was responsible for the intrinsic penicillin resistance [7].

The role of outer membrane protein (OMP) in antibiotic resistance is usually related to permeation [8] as antibiotic susceptibility is related to OMP channel size [9]. Ampicillin enters *E. coli* through OmpF [10]. In carbapenem-resistant*Enterobacter aerogenes*, the expression of OMPs is deficient due to overexpressed sRNA or decreased due to single-point mutation [11]. In addition, performing gene knockout in *V. parahaemolyticus* is challenging, so few studies have reported antibiotic resistance in OMP mutants.

In this work, *VP_RS22195*, *VP_RS23020*, *VP_RS16800*, *VP_RS16465*, *VP_RS20840*, *VP_RS03765*, and *VP_RS11205* were knocked out by ATCC33846. Subsequently, the growth, outer membrane (OM) permeability, and minimum inhibitory concentration (MIC) of OMP deletion mutants were evaluated. In total, 14 antibiotics targeting different sites in cells were selected, including *β*-lactams (ampicillin), aminoglycosides (streptomycin, kanamycin, gentamicin, tobramycin), quinolones (ciprofloxacin, nalidixic acid), furans (nitrofurantoin), cationic antimicrobial peptides (polymyxin B), rifampicin, clarithromycin, tetracycline, chloramphenicol, and novobiocin. After ampicillin treatment, the transcriptional changes of 20 genes in each mutant were further detected. This study gives a better understanding of the role of OMPs in *V. parahaemolyticus* OM and the cell wall.

## 2. Materials and Methods

### 2.1. Strain and Growth Condition

Table 1 lists all strains and plasmids used in this study. *V. parahaemolyticus* ATCC33846 was used to construct OMP deletion strains. The bacteria were grown in Luria-Bertani (LB) broth medium containing 10 g/L NaCl, 10 g/L trypsin (OXOID), and 5 g/L yeast extract (OXOID) at 37°C and 200 rpm for liquid culture.

### 2.2. Construction of Deletion Plasmid pOTC-SB

The deletion plasmid pOTC-SB was composed of genes *CmR*, *p15A*, *traJ*, *oriT*, and *cre*. The *CmR* and *p15A* gene fragment (1) was amplified from template pACYC184 with primers *Cm*-*p15A*-F and *Cm*-*p15A*-R, and then digested with FastDigest enzymes *Spe*I and *Pst*I (Thermo Scientific). The *traJ* and *oriT* gene fragment (2) was amplified from template pDS132 with primers *traJ*-*oriT*-F and *traJ*-*oriT*-R and then digested with FastDigest enzymes *Sal*I and *Spe*I (Thermo Scientific). The *cre* gene fragment (3) was amplified from template pDTW109 by primers P*tac*-*cre*-F and P*tac*-*cre*-R. The *sacB* gene fragment was amplified from template pDS132 by primers *sacB*-F and *sacB*-R. Finally, the ligation product of (1) and (2) was connected to (3) by using one-step cloning (ClonExpress II One Step Cloning Kit, Vazyme). The resulting plasmid was named pOTC as it contained *oriT* and *cre*. Linearized pOTC by *Bst*Z17I and *sacB* were combined by one-step cloning to construct pOTC-SB.

### 2.3. Construction of OMP Deletion Mutants

Upstream and downstream homology arms were amplified from the chromosomal DNA of *V. parahaemolyticus* ATCC33846 by the corresponding U/D-(*target gene*)-F/R primers, as displayed in Table 2. The *loxL*-*Gm*-*loxR* fragment was amplified from pWJW101 [15] by primers *Gm*-R and *Gm*-F, and the homology arms and *loxL-Gm-loxR* were integrated by fusion PCR (4). Then, the pDS132 plasmid and (4) were separately digested with the FastDigest enzymes *Xba*I or *Sal*I and ligated by T4 ligase (*VP_RS23020* was connected to pDS132 by one-step cloning). *E. coli* CC118 was transformed with products and cultured on LB plates containing 30 *μ*g·ml^−1^ gentamicin, and the plasmid extracted from *E. coli* CC118 was electrotransformed into *E. coli*S17-l (*λpir*).

After conjugation of *V. parahaemolyticus* ATCC33846 and *E. coli*S17-l (*λpir*) with the relevant plasmid, 5 *μ*g·ml^−1^ polymyxin B and 10 *μ*g·ml^−1^ chloramphenicol were used to select transformants of *V. parahaemolyticus*. Then, 10% (W/V) sucrose and gentamicin (10 *μ*g·ml^−1^) were used to select deletion mutants. The mutants were verified by colony PCR and genome verification.

**Figure 1:**
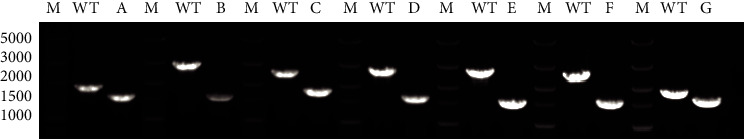
The verification of OMP deletion mutants was performed using primers of (gene)-U-F and (gene)-D-R by DNA agarose gel electrophoresis. M marker; WT, wild type, representing ATCC33846; lanes A-H showed Δ*VP_RS22195*, Δ*VP_RS23020*, Δ*VP_RS16800*, Δ*VP_RS16465*, Δ*VP_RS20840*, Δ*VP_RS03765*, and Δ*VP_RS11205* respectively.

Following knockout, *Gm* was removed by Cre in pOTC-SB. The deletion mutants were conjugated with S17-1 containing pOTC-SB and then spread-plated with 30 *μ*g·ml^−1^ chloramphenicol. Agarose gel electrophoresis revealed three results. The first result indicated a single band with the length of homology arms plus *Gm*; the second result showed a single band with the length of homology arms plus 100 bp; the third result displayed a combination of the above two types of bands. To ensure thorough Cre, the strains of the first and third colonies were cultured again in LB with chloramphenicol until the second result appeared. Then, negative selection was performed on an LB plate with gentamicin to ensure the removal of *Gm* from the genome by Cre. The pOTC-SB was removed by culturing in LB with 10% sucrose without antibiotics. The negative selection was performed on an LB plate with chloramphenicol to verify the removal of pOTC-SB, which has chloramphenicol resistance. The verification of OMP deletion mutants by agarose gel electrophoresis is shown in Figure 1.

After OMP deletion mutants were constructed, mutants and wild-type strains were streaked and purified on the LB plate 5 times, then cultured in LB liquid overnight and stored at −70°C. Before each assay, strains were streaked and activated on the LB plate for 25 h, and then a single colony was cultured in LB liquid for 14 h. A bacterial solution was then used in each assay.

### 2.4. Growth Curve

The overnight cultured strains were added to 50 ml of LB broth medium with an initial OD_600_ of 0.02. The mixture was cultured at 37°C and 200 rpm. All assays were performed in triplicate.

### 2.5. Permeation Assay

The OD_600_ of overnight-cultured strains was adjusted to 0.5. The sediments were washed twice and suspended in 10 mmol·L^−1^ pH 7.4 PBS. Subsequently, 48 *μ*l NPN solution was added to 1152 *μ*l of the bacteria solution before being observed with the fluorescence spectrophotometer. The detection was carried out under excitation light at 350 nm, emission light at 428 nm, and a 2.5 mm slit width. All assays were carried out in triplicates, and three parallels were performed in each group.

### 2.6. The Minimum Inhibitory Concentration (MIC) Assay

All antibiotics except ampicillin were diluted by a two-fold dilution method to adjust the concentration to 0.0078125–256 *μ*g·ml^−1^ in 96-well plates. As the 2-fold dilution of high-concentration solutions would result in large intervals, another concentration setting was used for ampicillin. For solutions with a concentration under 100 *μ*g·ml^−1^, a 2-fold dilution method was used to achieve a concentration range of 0.98–125 *μ*g·ml^−1^. For solutions with a concentration range of 100–1000 *μ*g·ml^−1^, the resulting concentration was set to 100, 200, 300,…, 1000 *μ*g·ml^−1^. In the concentration range between 1000 and 2000 *μ*g·ml^−1^, the concentration points were set as 1000, 1250, 1500, and 1750 *μ*g·ml^−1^. In addition, for concentrations above 2000 *μ*g·ml^−1^, final concentrations of 2000, 2500, and 3000 *μ*g·ml^−1^ were set up. The strains cultured overnight were then transferred to 5 ml LB test tubes with an initial OD_600_ of 0.02 for 4-5 h. Then, the OD_600_ of freshly cultured strains was adjusted to 0.001. Then, 100 *μ*l of diluted bacteria solution was added to each well, and the culture was incubated at 37°C for 18 h. OD_600_ was measured by BioTek Cytation 5. All assays were carried out in duplicate and performed three times in parallel in each group.

### 2.7. Real-Time Polymerase Chain Reaction (RT-qPCR)

The overnight cultured strains were transferred into 5 ml LB test tubes with an initial OD_600_ of 0.02 for 4–5 h. Half of the bacterial solution was taken out into a sterilized empty test tube. Ampicillin was added to the experimental groups at the final concentration of 16 *μ*g·ml^−1^, and sterilized water with the same volume of ampicillin was added to tubes as the control. After incubating at 37°C and 200 rpm for 0.5 h, the sediment was used for RNA extraction. The methods used for RNA extraction, reverse transcription, and RT-qPCR were the same as those described in [17]. All assays were performed in triplicates.

## 3. Results

### 3.1. Deletion of OMP Affects Cell Growth, OM Permeation, and Antibiotic Resistance

**Figure 2:**
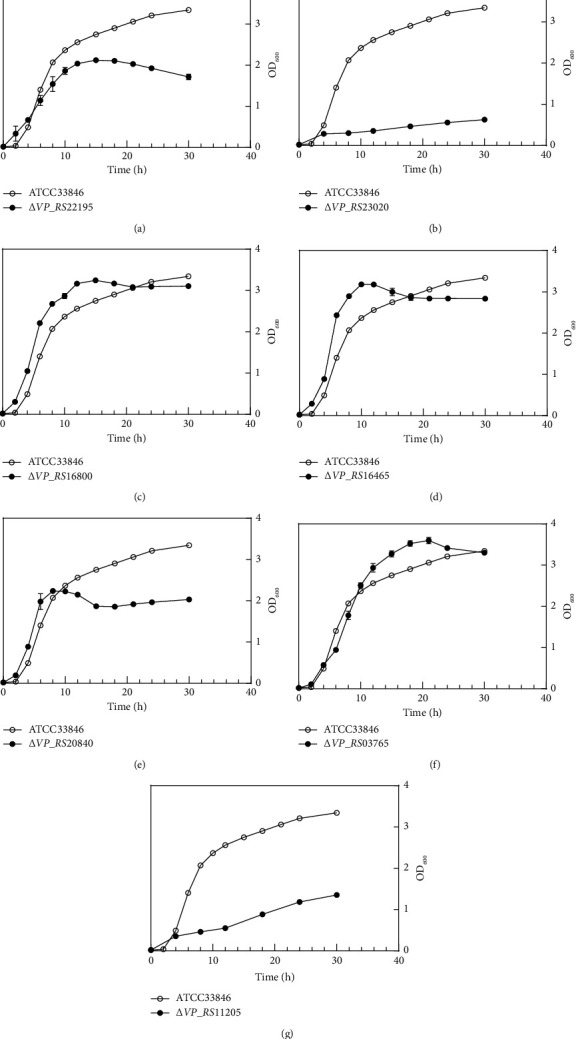
Growth curves of the 7 *V. parahaemolyticus* OMP deletion mutants. Wild-type*V. parahaemolyticus* ATCC33846 was used as a control. Each point represents the mean value of three biological replicates, and error bars represent standard deviations calculated from three biological replicates.

Bacterial growth has four phases, including the lag phase, the exponential phase, the stationary phase, and the decline phase. In the lag phase, bacteria produce new enzymes to digest, build biomass, and prepare for cell division [18]. ATCC33846 started to grow after a lag phase of 2 hours following the inoculum. Compared to ATCC33846, some OMP mutants demonstrated a shorter lag phase, including Δ*VP_RS22195*, Δ*VP_RS16800*, Δ*VP_RS16465*, and Δ*VP_RS20840* (Figure 2). The shorter lag phase indicated that the OMPs were the proteins prepared in the lag phase. Deletion of OMPs lightened the burden of preparation for cells in the lagged phase. In the exponential phase, Δ*VP_RS16800*, Δ*VP_RS16465*, Δ*VP_RS20840*, and Δ*VP_RS03765* (Figures 2(c)–2(f)) had a higher growth rate than ATCC33846. Furthermore, Δ*VP_RS22195*, Δ*VP_RS16800,* Δ*VP_RS16465*, Δ*VP_RS20840*, and Δ*VP_RS03765* entered the decline phase directly, without an obvious stationary phase. In contrast, Δ*VP_RS23020* and Δ*VP_RS11205* showed poor growth (Figures 2(b) and 2(g)).

**Figure 3:**
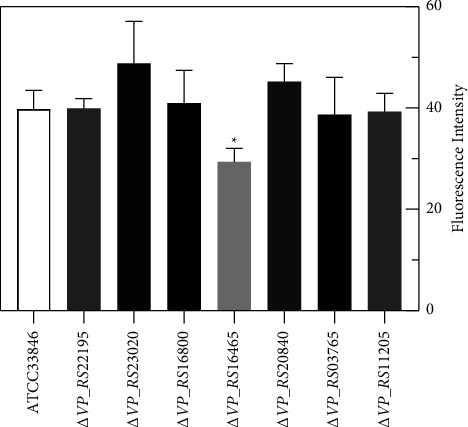
Comparison of OM permeability of the 7 *V. parahaemolyticus* OMP mutants, using wild-type ATCC33846 as a control. The fluorescence intensity was determined with excitation at 350 nm and emission at 428 nm. Each bar represents the mean value of three biological replicates, and error bars represent standard deviations calculated from three biological replicates. A statistically significant difference was determined using independent samples *t*-test analysis (^*∗*^*P*  < 0.05).

The N-Phenyl-1-naphthylamine (NPN) probe was used to test the OM permeability of OMP deletion mutants. NPN can be excited to form green fluorescence in the bacterial inner membrane, a hydrophobic environment [19]. A decreased OM permeability was observed in Δ*VP_RS16465* (Figure 3).

**Figure 4:**
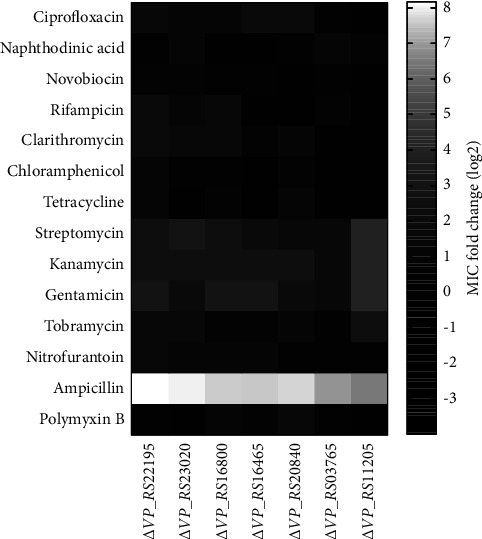
Comparison of resistance to 14 antibiotics among the 7 *V. parahaemolyticus* OMP deletion mutants, using wild-type ATCC33846 as a control. The fold change is calculated by log_2_ (the MIC of mutants/MIC of ATCC33846). Each color block represents the mean value of three biological replicates.

Fourteen antibiotics were selected for the MIC assay (Figure 4). In Δ*VP_RS23020*, increased resistance to antibiotics (ciprofloxacin, nalidixic acid, and novobiocin) inhibiting DNA synthesis was observed. Rifampicin inhibits RNA synthesis, and a 4-fold increase in the rifampicin MIC was observed in Δ*VP_RS22195*. Resistance to antibiotics (clarithromycin, chloramphenicol, tetracycline, streptomycin, kanamycin, gentamicin, and tobramycin) inhibiting protein synthesis was all increased in Δ*VP_RS22195* and Δ*VP_RS20840*. Among protein synthesis-inhibiting antibiotics, resistance to 3 aminoglycosides (streptomycin, kanamycin, and gentamicin) was increased, whereas the fold change of tobramycin MIC was not obvious. Notably, Δ*VP_RS11205* was more resistant to aminoglycosides than other mutants, and the tetracycline susceptibility of Δ*VP_RS23020* was increased 2-fold. Nitrofurantoin inhibits carbohydrate metabolism enzymes and interferes with cell wall synthesis [20]. Resistance to nitrofurantoin was not decreased after OMP deletion. In addition, resistance to *β*-lactams, which act on peptidoglycan (PG) synthesis, showed a general increase. Polymyxin B damages Gram-negative bacterial OM and resistance to polymyxin B was increased 2.8-fold in Δ*VP_RS20840*. All OMP mutants showed at least a 64-fold increase in ampicillin resistance.

### 3.2. Changes in the Transcription of 20 Genes in OMP Deletion Mutants under the Absence and Stimulation of Ampicillin

RT-qPCR was performed to detect the transcriptional changes of 20 genes to further study the causes of increased ampicillin resistance in OMP deletion mutants (Table 3). These genes can be divided into five types: OMP genes, *β*-lactamase genes, PG synthesis-related genes, stress-regulation-related genes, and lipid A synthesis genes. *VP_RS17515* expresses *β*-lactamase. *VP_RS13510*, *mrcB*, *mrdA*, *VP_RS02165*, *VP_RS03450*, *VP_RS22785*, *dacB*, *VP_RS22200*, *VP_RS09310*, *VP_RS15980* were selected as PG synthesis-related genes through BLASTp in NCBI, and the proteins were homologous with PBP1A, PBP1B, PBP2, PBP3, PBP5, PBP4, LdtA, LdtD, and LdtF, respectively, in *E. coli*. In order to directly observe gene regulation under membrane stress, *uhpA* and *VP_RS14060*, which are homologous to the response regulator proteins *rcsB* and *cpxR* in *E. coli,* respectively, were selected. *lpxA* is a lipid A synthesis gene. *β*-lactams inhibit bacterial growth by binding to penicillin-binding proteins (PBPs) and interfering with PG synthesis [27]. According to the growth curve, bacteria in the initial exponential phase were used as the experiment sample. The ampicillin MIC value of ATCC33846 was 3.90625–15.625 *μ*g·mL^−1^, while the MIC values of OMP mutants were above 600 *μ*g·mL^−1^. Therefore, 16 *μ*g·mL^−1^ ampicillin, which would not kill ATCC33846 and have a better effect on mutants, was set as the pretreatment.

**Figure 5:**
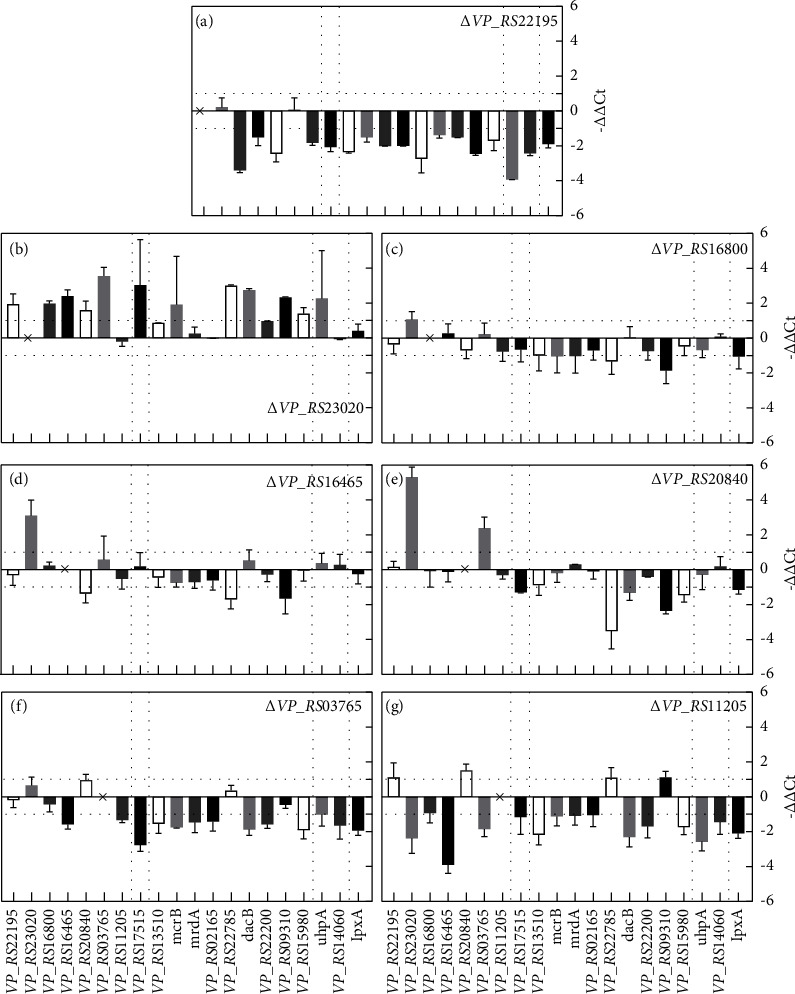
Comparison of transcription of 20 genes in the 7 *V. parahaemolyticus* OMP deletion mutants, using wild-type ATCC33846 as a control. Five kinds of genes are divided by dotted line: OMP genes, *β*-lactamase genes, PG synthesis-related genes, stress-regulation-related genes, and lipid A synthesis genes. *VP_RS17515* expresses *β*-lactamase. The transcription change fold is calculated by 2^−ΔΔCt^. Each bar represents the mean value of three biological replicates, and error bars represent standard deviations calculated from three biological replicates.

Figure 5 shows the transcription changes of 20 genes in mutants compared to ATCC33846. In Δ*VP_RS23020* (Figure 5(b)), all genes exhibited transcriptional upregulation except for *VP_RS11205*. In Δ*VP_RS16465* and Δ*VP_RS20840* (Figures 5(d) and 5(e)), *VP_RS23020* was upregulated over 9-fold. The transcription of *VP_RS17515* was also upregulated over 9-fold in Δ*VP_RS23020* but downregulated in Δ*VP_RS22195*, Δ*VP_RS16800*, Δ*VP_RS20840*, Δ*VP_RS03765*,and Δ*VP_RS11205* (Figures 5(a), 5(c), 5(e)–5(g)). Most PG synthesis genes were downregulated in mutants except for Δ*VP_RS23020*. However, in Δ*VP_RS23020*, the transcription of *VP_RS22200*, *VP_RS09310*, and *VP_RS15980* (*Ldt*s) were all upregulated. Among PG synthesis genes, the transcriptional changes of *VP_RS22785* and *VP_RS09310* showed an interesting phenomenon. These two genes were downregulated over 2-fold in Δ*VP_RS22195*, Δ*VP_RS16800*, Δ*VP_RS16465*, and Δ*VP_RS20840*. In contrast, they were upregulated over 2-fold in Δ*VP_RS23030* and Δ*VP_RS11205*, *VP_RS22785*, and *VP_RS09310*. In Δ*VP_RS03765*, these two genes showed no obvious downregulation compared to other genes. Moreover, *VP_RS20840* showed the same trend as *VP_RS22785* in all mutants. Moreover, the transcription of *lpxA* was downregulated in all mutants except for Δ*VP_RS23020*.

**Figure 6:**
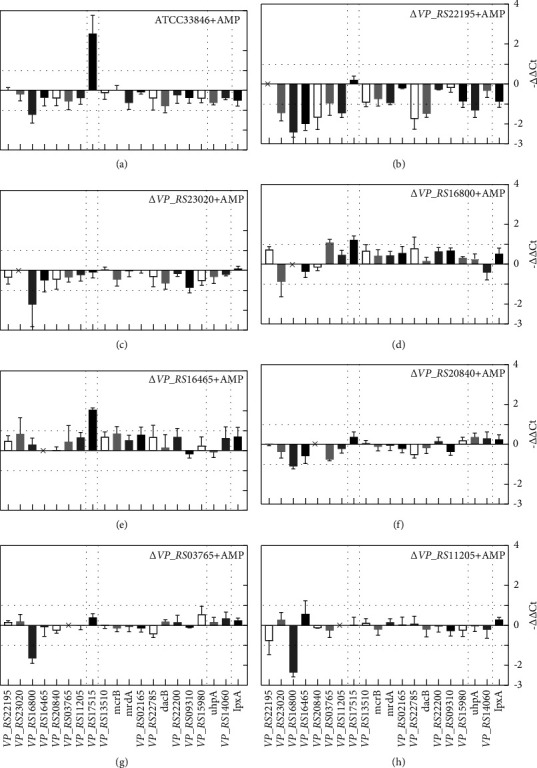
Comparison of transcription of 20 genes in the 7 *V. parahaemolyticus* OMP deletion mutants under stimulation of ampicillin (AMP), using wild-type ATCC33846 as a control. Five kinds of genes are divided by dotted line: OMP genes, *β*-lactamase genes, PG synthesis-related genes, stress-regulation-related genes, and lipid A synthesis genes. The transcription change fold is calculated by 2^−ΔΔCt^. Each bar represents the mean value of three biological replicates, and the error bars represent standard deviations calculated from three biological replicates.

Figure 6 displays the transcription changes of OMP genes of ATCC33846 and mutants under ampicillin stimulation compared to the untreated group. All OMP genes were downregulated in Δ*VP_RS22195*, Δ*VP_RS23020*, and Δ*VP_RS20840* (Figures 6(b)6(c), and 6(f)). Furthermore, *VP_RS16800* was downregulated over 2-fold in ATCC33846, Δ*VP_RS23020*, Δ*VP_RS20840*, and Δ*VP_RS03765* (Figures 6(a), 6(c), 6(f), and 6(g)), and over 4-fold in Δ*VP_RS22195* and Δ*VP_RS11205* (Figures 6(b) and 6(h)). *VP_RS17515* demonstrated over 2-fold upregulation in ATCC33846, Δ*VP_RS16800*, and Δ*VP_RS16465* (Figures 6(a), 6(d), and 6(e)). In addition, transcription of PG synthesis genes showed significant changes in OMP deletion mutants but not after ampicillin stimulation. These findings implied that OMP deletion caused internal resistance to ampicillin instead of inducing a stress response with stimulation of ampicillin.

## 4. Discussion

The MIC change folds showed two major increases in resistance to aminoglycosides and ampicillin (Figure 4), and the MIC value is shown in Table S1. The MIC values of 3 kinds of aminoglycosides (streptomycin, kanamycin, and gentamicin) increased in all 7 OMP mutant strains, indicating that the mechanism of aminoglycoside resistance could be related to OM. Aminoglycosides act on bacterial ribosomes and inhibit translation [28]. Moreover, aminoglycosides are polycationic at physiological pH and can replace divalent cations on lipopolysaccharide, thereby increasing membrane permeability [29]. However, the results of the MIC fold change of polymyxin B, which could also damage OM by replacing divalent cations on lipopolysaccharides, showed that not all the OM of mutants was more resistant to cationic antibiotics. In addition, after the deletion of OMP genes, the fold change of tobramycin's MIC was lower than that of other aminoglycosides, and Δ*VP_RS03765* was sensitive to tobramycin. Therefore, the aminoglycoside resistance mechanism remains unclear in OMP deletion mutants.

A significant increase in ampicillin resistance was observed, with ATCC33846 showing a small MIC value (fluctuating 3.90625–15.625 *μ*g·mL^−1^). However, 10.6% of *V. parahaemolyticus* isolates from the coast of Korea were sensitive to ampicillin, while 87.2% were resistant [30]. It is speculated that the low ampicillin MIC of ATCC33846 was caused by repeated subculturing through different generations without antibiotics in the laboratory and an ATCC33846 sample at the early exponential phase. In addition, different CARB *β*-lactamases in various *V. parahaemolyticus* strains exhibit different ampicillin hydrolysis rates [31].

Ampicillin resistance in different OMP mutants may result from reduced PG synthesis activity and expression of *β*-lactamase. PG synthesis can be affected by reduced transcription of PBPs, increased transcription of Ldts, downregulated D,d-carboxypeptidase, and alanine deficiency.

Reduced transcription of PBPs was inferred to be one cause of ampicillin resistance. In Δ*VP_RS22195*, Δ*VP_RS03765*,and Δ*VP_RS11205*, PG synthesis-related genes were downregulated (Figures 5(a), 5(f), and 5(g)). *VP_RS22195* is located adjacent to *VP_RS22200* in the genome and has 40.45% protein similarity to the murein lipoprotein Lpp in *E. coli* (Table 3). Furthermore, the N-terminus of lipoprotein LpoB is required for the activation of PBP1B in *E. coli* [32]. Lipoprotein NlpI is a part of PG biosynthetic multienzyme complexes and acts as an adaptor [33]. In addition, transcription of *uhpA* in Δ*VP_RS22195* was downregulated over 15-fold (Figure 5(a)). The lack of lipoprotein increases the periplasmic distance, and OM stress signals cannot be transmitted by the Rcs system [34]. PG synthesis was affected after the deletion of *VP_RS22195*, resulting in a slower division rate in the exponential phase than ATCC33846 (Figure 2(a)). Another cause might be the upregulated Ldts in Δ*VP_RS23020*. In a *β*-lactam-resistant mutant of *Enterococcus faecium*, Ldt_fm_ was found to account for *β*-lactam resistance by using a different substrate from d,d-transpeptidase [6].

Δ*VP_RS16465* and Δ*VP_RS20840* might exhibit ampicillin resistance due to the downregulated transcription of *VP_RS22785* (Figures 5(d) and 5(e)), which encodes a D,d-carboxypeptidase cleaving pentapeptides into tetrapeptides [23]. The *V. cholerae*d,d-endopeptidase ShyA could recognize but not cleave dimers containing pentapeptides [35]. Therefore, PG was protected from being cleaved by d,d-endopeptidases, and existed PG was maintained, reducing the activity of PG synthesis to resist ampicillin. In addition, with lower tetrapeptides, the transcription of *VP_RS09310* was downregulated, which is homologous to l,D-transpeptidase LdtD cleaving tetrapeptide to form mDAP^3^–mDAP^3^cross-links (Table 3). However, the deletion of PBP5 in *E. coli* increased the sensitivity to *β*-lactams [36]. It is speculated that 3 homologs of PBP5 in *V. parahaemolyticus* and one of the downregulated homologs do not exert a significant impact on maintaining the normal cell shape. In addition, the OMP gene *VP_RS20840* showed the same trend in transcriptional change as *VP_RS22785* in all OMP mutants (Figure 5). This finding implies an unknown relationship between *VP_RS22785* and *VP_RS20840*. In addition, alanine deficiency may be involved in increased ampicillin resistance. Previous research reported lysis in an *E. coli* strain lacking alanine racemase in the absence of D-ala, which is mainly caused by defects in PG synthesis [37]. OmpA was upregulated after the addition of alanine through analysis of proteomics and RT-qPCR [38]. It is speculated that lower levels of alanine entered the cell following OmpA deletion, which might further affect the transcription of PBP genes. *VP_RS16465*, *VP_RS20840*, and *VP_RS03765* belong to the OmpA protein family (Table 3), while the different transcription trends of *VP_RS22785* inferred different functions of OmpA.

The existence and expression of *β*-lactamase were one of the causes of ampicillin resistance in *V. parahaemolyticus*. After ampicillin treatment, *VP_RS17515* (*β*-lactamase) was upregulated in other strains except for Δ*VP_RS23020* (Figure 6(c)). The same conclusion was obtained in *V. parahaemolyticus* V110 [7]. Although the transcription of *VP_RS17515* was slightly downregulated in Δ*VP_RS23020* under ampicillin stimulation (Figure 6(c)), it was upregulated more than 4-fold in Δ*VP_RS23020* (Figure 5(b)). *VP_RS23020* encodes maltoporin. In addition, the maltose metabolism pathway was potentially involved in the resistance to antibiotics that target cell wall biosynthesis. In a *Lactococcus lactis* strain resistant to lactococcin 972, which is a bacteriocin that inhibits cell wall biosynthesis by binding to lipid II, maltose metabolic genes were deleted. However, this strain showed no lactococcin 972 sensitivity in the maltose medium [39].

Lipoprotein potentially affected OM biosynthesis through phospholipids. Transcription of *lpxA* was downregulated over 3.7-fold in Δ*VP_RS22195* (Figure 5(a)), indicating that the lack of VP_RS22195 had an effect on OM synthesis. The maturity of lipoprotein is associated with phosphatidylglycerol [40]. Moreover, crosstalk between phospholipids and lipopolysaccharide synthesis was observed. LpxK catalyzes the synthesis of lipid IV A from lipid A disaccharide, which depends on the concentration of unsaturated fatty acids [41]. Furthermore, transcription of *lpxA* was downregulated over 2-fold in Δ*VP_RS16800*, Δ*VP_RS20840*, Δ*VP_RS03765*, and Δ*VP_RS11205*. Nevertheless, the relationship between these OMPs and OM synthesis remains unknown.

## 5. Conclusions

Deletion of OMP affects growth and OM permeation, and MIC and OMP mutants demonstrated significantly increased ampicillin resistance. Further RT-qPCR analysis showed several possible causes of ampicillin resistance in OMP mutants, including the expression of *β*-lactamase, the reduction of PG synthesis activity due to reduced transcription of PBPs, increased transcription of Ldts, downregulated D,D-carboxypeptidase, and alanine deficiency. This study provides a new perspective on ampicillin resistance in OMP mutants with respect to PG synthesis. Future work will focus on the role of OMPs in the synthesis of OM and PG.

## Figures and Tables

**Table 1:** Bacteria and plasmids are used in this study.

Strains or plasmids	Descriptions	Sources
*Strains*		
ATCC33846	Wild-type*Vibrio parahaemolyticus*	ATCC
CC118(*λ*pir)	*λ*-pir lysogen of CC118 (D(ara-leu) araD DlacX 74 galE galK phoA20 thi-1rpsE rpoB argE(Am) recA1)	[12]
S17-1(*λ*pir)	*λ*-pir lysogen of S17-1 (thi pro hsdR–hsdM + recA RP4 2-Tc::Mu-Km::Tn7 (Cm^r^))	[13]
ATCC33846Δ*VP_RS22195*	Derived from ATCC33846 by removing *VP_RS22195*	This study
ATCC33846Δ*VP_RS23020*	Derived from ATCC33846 by removing *VP_RS23020*	This study
ATCC33846Δ*VP_RS16800*	Derived from ATCC33846 by removing *VP_RS16800*	This study
ATCC33846Δ*VP_RS16465*	Derived from ATCC33846 by removing *VP_RS16465*	This study
ATCC33846Δ*VP_RS20840*	Derived from ATCC33846 by removing *VP_RS20840*	This study
ATCC33846Δ*VP_RS03765*	Derived from ATCC33846 by removing *VP_RS03765*	This study
ATCC33846Δ*VP_RS11205*	Derived from ATCC33846 by removing *VP_RS11205*	This study

*Plasmids*		
pDS132	Suicide plasmid, Cm^r^	[14]
pACYC184	Plasmid containing *p15A* origin, Cm^r^, Tc^r^	ATCC
pWJW101	Plasmid containing *loxL-Gm-loxR*, Gm^r^	[15]
pDTW109	Plasmid containing *cre* gene, Gm^r^	[16]
pΔ*VP_RS22195*	Derived from pDS132 by adding *VP_RS22195* homologous arms	This study
pΔ*VP_RS23020*	Derived from pDS132 by adding *VP_RS23020* homologous arms	This study
pΔ*VP_RS16800*	Derived from pDS132 by adding *VP_RS16800* homologous arms	This study
pΔ*VP_RS16465*	Derived from pDS132 by adding *VP_RS16465* homologous arms	This study
pΔ*VP_RS20840*	Derived from pDS132 by adding *VP_RS20840* homologous arms	This study
pΔ*VP_RS03765*	Derived from pDS132 by adding *VP_RS03765* homologous arms	This study
pΔ*VP_RS11205*	Derived from pDS132 by adding *VP_RS11205* homologous arms	This study

**Table 2:** Primers used in this study.

Names	Sequence (5′–3′)
*Ptac*-*cre*-F	TTGGATACACCAAGGAAAGTGGTACCTGAACGACCCCGAATATTGG
*Ptac*-*cre*-R	ACAGATGTAGGTGTTCCACACTGCAGCTAATCGCCATCTTCCAGCA
*traJ*-*oriT*-F	C**ACTAGT**TCGGGTCGGGTGAATCTT
*traJ*-*oriT*-R	ACTTTCCTTGGTGTATCC
*CmR*-*p15A*-F	TGTGGAACACCTACATCTG
*CmR*-*p15A*-R	C**ACTAGT**ATCGTATGGGGCTGACTT
*sacB-*F	CAGCGCTAGCGGAGTGTATACCACCTTTATGTTGATAAGAAATAAAAGAAA
*sacB-*R	CAACATAGTAAGCCAGTATACGGATCGATCCTTTTTAACCCATC
*GmR*-R	CGTAATACGACTCACTATAGGGC
*GmR*-F	GCGCAATTAACCCTCACTAAAG
*VP_RS22195*-U-F	AA**TCTAGA**CAGAATCACAGCACGAGG
*VP_RS22195*-U-R	GCCCTATAGTGAGTCGTATTACGCTCCCACAACAGTCCACATAGC
*VP_RS22195*-D-F	CTTTAGTGAGGGTTAATTGCGCCTAAGTAAGATGTTGTTCCGCG
*VP_RS22195*-D-R	AA**TCTAGA**TGGCAGCGAACAAAGGTG
*VP_RS23020*-U-F	GGGTTAAAAAGGATCGATCCTGTCGTTTTTCGCAACAATC
*VP_RS23020*-U-D	GCCCTATAGTGAGTCGTATTACGAAAATGGCTTGCTCCCGT
*VP_RS23020*-D-F	CTTTAGTGAGGGTTAATTGCGCAGCACGCATGTAACCGTG
*VP_RS23020*-D-R	AATTTGTGGAATTCCCGGGAGTATGACGTATACCCGCATA
*VP_RS16800*-U-F	AA**TCTAGA**CCTCTTTTGGCTCGGGTT
*VP_RS16800*-U-R	GCCCTATAGTGAGTCGTATTACGAAACGGGCTAACTACTGT
*VP_RS16800*-D-F	CTTTAGTGAGGGTTAATTGCGCCGTTACGGATGTATGTGT
*VP_RS16800*-D-R	AA**TCTAGA**GACACTGAACTGCCTGTC
*VP_RS16465*-U-F	AA**TCTAGA**AAGACGAACCAGGCAAGG
*VP_RS16465*-U-R	GCCCTATAGTGAGTCGTATTACGAGTGTCAGCAAGTGCAAC
*VP_RS16465*-D-F	CTTTAGTGAGGGTTAATTGCGCCGATGCTCCGTTTAGTAG
*VP_RS16465*-D-R	AA**TCTAGA**GAATGGCTAGTAAGGAATGT
*VP_RS20840*-U-F	AAA**GTCGAC**GAAGACCGTCATTTAGCT
*VP_RS20840*-U-R	GCCCTATAGTGAGTCGTATTACGGCAAGTCTATGGGGAAGC
*VP_RS20840*-D-F	CTTTAGTGAGGGTTAATTGCGCCATTCTCATCAAAAGGGG
*VP_RS20840*-D-R	AAA**GTCGAC**GCCATACGGAGGATTCAT
*VP_RS03765*-U-F	AA**TCTAGA**AAATCGGTATGCCACTTC
*VP_RS03765*-U-R	GCCCTATAGTGAGTCGTATTACGGCCGCCTAATAACACTGAC
*VP_RS03765*-D-F	CTTTAGTGAGGGTTAATTGCGCGACCGTAGAATAGCAGCC
*VP_RS03765*-D-R	AA**TCTAGA**CATCGCTCAGTGGGTTT
*VP_RS11205*-U-F	AA**TCTAGA**CGCCTCTATCGTTCGCAT
*VP_RS11205*-U-R	GCCCTATAGTGAGTCGTATTACGGACAATTACCGGGGGTGAG
*VP_RS11205*-D-F	CTTTAGTGAGGGTTAATTGCGCCCTCTTTAGAAAGTTCTACCG
*VP_RS11205*-D-R	AA**TCTAGA**TTCAACATCTCTTGGACTC
RT-*VP_RS22195*-F	CTAAGCAACCAAGTTAGCCAAC
RT-*VP_RS22195*-R	TTCTTCTTGAGCAGCCATTG
RT-*VP_RS23020*-F	AGTTGAATCTCGCCGTAAAT
RT-*VP_RS23020*-R	CTGCTGGTTCTGCTTTCG
RT-*VP_RS16800*-F	TGCTACATCGCCATCACG
RT-*VP_RS16800*-R	GCATCAACTACCGCTTCTTG
RT-*VP_RS16465*-F	GCTGGTGACGAGAAAGACT
RT-*VP_RS16465*-R	TTGAAGCCGATACCGAAG
RT-*VP_RS20840*-F	CAACAGGCTCACTAGGTGCT
RT-*VP_RS20840*-R	TTTGCGTCCATATCGTCC
RT-*VP_RS03765*-F	CTCAAACTATCGGCACTGGT
RT-*VP_RS03765*-R	TTCGCTTGTGGGTGCTC
RT-*VP_RS11205*-F	CGTAAGCGATTTTCTGTGC
RT-*VP_RS11205*-R	AAAGCGGCTGGGATTGG
RT-*VP_RS17515*-F	GCTTGTCCGTTTGTGTATCCC
RT-*VP_RS17515*-R	TGCTCAACTGTTAGTTACGCCTC
RT-*VP_RS13510*-F	AATCATTGCTCGTTACCACAG
RT-*VP_RS13510*-R	CCGACGTATAGGCTTTCTCTTC
RT-*mrcB*-F	GCGACAGAAGACCGAGAT
RT-*mrcB*-R	CGTTAAGGTACTGCCACCT
RT-*VP_RS02165*-F	TCGCTTACCGTGCCATC
RT-*VP_RS02165*-R	TTTTACATCCAGCATCACCAC
RT-*mrdA*-F	GTTTTGATGGGCTTGCTG
RT-*mrd*-R	CCACTTTGATGCGGTTGT
RT-*VP_RS21250*-F	TTGACGGGATGAAAACGG
RT-*VP_RS21250*-R	AACAACTGCGATAAGGCG
RT-*VP_RS22785*-F	TGGCATCCAAACCTCACC
RT-*VP_RS22785*-R	CTTGAAGACTTTCTCGCTGT
RT-*dacB*-F	AGACCAACTATTTCCACCTG
RT-*dacB*-R	AGTAGATAACGGCATCGG
RT-*VP_RS22200*-F	ACGCCACCTGCGTCTATT
RT-*VP_RS22200*-R	CATTCCGATGCCGAAATC
RT-*VP_RS09310*-F	TAATGACGGCACTTATGGT
RT-*VP_RS09310*-R	ACAGACAACTGCTCTACGG
RT-*VP_RS15980*-F	AGAAGAAGGCGACAACCG
RT-*VP_RS15980*-R	GCACCGAGCGATAAAACG
RT-*uhpA*-F	AAGGCTATCTAAGCAAACGC
RT-*uhpA*-R	GCAATGTCGGAGGTAAGGT
RT-*VP_RS14060*-F	TAGAGCTCGGCGCAGATG
RT-*VP_RS14060*-R	TTGCTTGCCCGGATACAG

*Note.* The restriction enzyme sites are in bold.

**Table 3:** Introduction of genes related to this work.

Gene names	Descriptions
*VP_RS22195*	Hypothetical protein, 438 bp, chromosome 2
	40.45% similar to murein lipoprotein Lpp in *Escherichia coli*
*VP_RS23020*	Maltoporin, 1311 bp, chromosome 2
	72.36% similar to maltoporin in *Escherichia coli*
*VP_RS16800*	MipA/OmpV family protein, 781 bp, chromosome 2
	100% similar to MipA/OmpV family protein in *Escherichia coli*
*VP_RS16465*	OmpA family protein, 1080 bp, chromosome 2
	28.77% similar to porin OmpA in *Escherichia coli*
*VP_RS20840*	OmpA family protein, 1318 bp, chromosome 2
	29.62% similar to porin OmpA in *Escherichia coli*
*VP_RS03765*	OmpA family protein, 1019 bp, chromosome 1
	32.5% similar to murein porin OmpA in *Escherichia coli*
*VP_RS11205*	OmpH family outer membrane protein, 465 bp, chromosome 1
	36.42% similar to periplasmic chaperone Skp in *E. coli*
*VP_RS17515*	Carbenicillin-hydrolyzing class A beta-lactamase CARB-22
	Hydrolyzing *β*-lactams [21]
*VP_RS13510*	PBP1A family penicillin-binding protein
	Peptidoglycan synthase which is essential for cell elongation [22]
*mrcB*	Penicillin-binding protein 1B
	Peptidoglycan synthase which is essential for cell division [22]
*mrdA*	Penicillin-binding protein 2
	Transpeptidase essential for cell elongation [23]
*VP_RS02165*	Penicillin-binding protein 3
	Transpeptidase essential for cell division [23]
*VP_RS22785*	D-alanyl-D-alanine carboxypeptidase
	D,D-carboxypeptidase which cleaves terminal D-ala in the peptidoglycan [24]
*dacB*	Serine-typeD-Ala-D-Ala carboxypeptidase
	D,D-carboxypeptidase which cleaves terminal D-ala in the peptidoglycan [23]
*VP_RS22200*	l,D-transpeptidase family protein
	Attaching Lpp to mDAP^3^ of peptidoglycan [23]
*VP_RS09310*, *VP_RS15980*	l,D-transpeptidase family protein
	Synthesizing mDAP^3^–mDAP^3^cross-links in the peptidoglycan [23]
*uhpA*	Transcriptional regulator, 29.06% similar to RcsB in *E. coli*
	RcsB is response regulator of Rcs sensing outer membrane stress [25]
*VP_RS14060*	Response regulator, 62.45% similar to CpxR in *E. coli*
	CpxR is response regulator of Cpx sensing inner membrane stress [26]
*lpxA*	Acyl-ACP-UDP-N-acetylglucosamine O-acyltransferase
	Essential for the biosynthesis of lipid A

## Data Availability

All the data generated or analysed during this study are included within the article.

## Supplementary Materials

Supplementary Materials Supplementary Table S1 shows the detailed data about MIC in Figure 4 and the MIC of amoxicillin.
