# Supplementary material for: The Influence of Outer Membrane Protein on Ampicillin Resistance of Vibrio parahaemolyticus
Source: Can J Infect Dis Med Microbiol. 2023 Jan 13;2023:8079091. doi: 10.1155/2023/8079091 (PMC9859689; doi:10.1155/2023/8079091)
Supplement: Supplementary Materials — Supplementary Table S1 shows the detailed data about MIC in Figure 4 and the MIC of amoxicillin. [file 8079091.f1.docx]

Table S1. 15 antibiotics MIC values of OMP deletion mutants.

| Antibiotics (μg·ml^-1^) | WT | Δ*VP_RS22195* | Δ*VP_RS23020* | Δ*VP_RS16800* | Δ*VP_RS16465* | Δ*VP_RS20840* | Δ*VP_RS03765* | Δ*VP_RS11205* |
| --- | --- | --- | --- | --- | --- | --- | --- | --- |
| Ciprofloxacin | 0.625 | 0.125~0.25 | 0.125 | 0.125 | 0.25 | 0.0625~1 | 0.03125~0.125 | 0.015625~0.03125 |
| Nalidixic acid | 2 | 2 | 4 | 1~2 | 1~2 | 2 | 4 | 2~4 |
| Novobiocin | 2~4 | 2~8 | 2~8 | 2~4 | 2~4 | 1~4 | 2~4 | 1~2 |
| Rifampicin | 0.03125~0.125 | 0.25 | 0.125 | 0.125~0.25 | 0.015625~0.125 | 0.015625~0.125 | 0.0625~0.125 | 0.015625~0.03125 |
| Clarithromycin | 2~4 | 8~16 | 8 | 8 | 4 | 2~16 | 1~4 | 1~4 |
| Chloramphenicol | 0.25~0.5 | 1~0.5 | 0.25~0.5 | 0.25~0.5 | 0.25 | 0.5 | 0.25 | 0.25 |
| Tetracycline | 0.5~1 | 1~2 | 0.25~0.5 | 0.5~2 | 0.5 | 1~2 | 0.25~1 | 0.25~1 |
| Streptomycin | 2~8 | 16~32 | 16~64 | 16~32 | 16 | 8~16 | 8~16 | 64 |
| Kanamycin | 2~4 | 16 | 8~32 | 8~32 | 16 | 16 | 8 | 32~64 |
| Gentamicin | 0.5~1 | 4~8 | 2~4 | 4~8 | 4~8 | 2~4 | 2 | 8~16 |
| Tobramycin | 2 | 4~8 | 4~8 | 2~4 | 2~4 | 4 | 1~4 | 8~16 |
| Nitrofurantoin | 0.5~1 | 2 | 1~2 | 1~2 | 1~2 | 0.25~2 | 0.5~1 | 0.25~2 |
| Ampicillin | 3.90625~15.625 | 1500~300 | 2000 | 1000~2500 | 1250~1750 | 1500~2000 | 800~1000 | 600~800 |
| Amoxicillin | 187.5~240 | 600~900 | Not detected | 500~800 | 500~700 | 500~700 | 400~450 | 300~400 |
| Polymyxin B | 8~32 | 16 | 8 | 32 | 16~32 | 32~64 | 16 | 8~16 |
